# When and how to discontinue bracing treatment in adolescent idiopathic scoliosis: results of a survey

**DOI:** 10.1186/s13013-018-0158-y

**Published:** 2018-10-26

**Authors:** Lucas Piantoni, Carlos A. Tello, Rodrigo G. Remondino, Ida A. Francheri Wilson, Eduardo Galaretto, Mariano A. Noel

**Affiliations:** Servicio de Patología Espinal, Hospital de Pediatría Prof. Dr. Juan P. Garrahan, Combate de los Pozos 1881, C1245AAM CABA Buenos Aires, Argentina

**Keywords:** Adolescent idiopathic scoliosis, Brace, Orthosis, Brace discontinuation, Non-operative scoliosis treatment

## Abstract

**Background:**

Currently, there is little consensus on how or when to discontinue bracing in adolescent idiopathic scoliosis (AIS). An expert spine surgeon national survey could aid in elucidate discontinuation of the brace.

Few data have been published on when and how to discontinue bracing treatment in patients with AIS resulting in differences in the management of the condition. The aim of this study was to characterize decision-making of surgeons in the management of bracing discontinuation in AIS.

**Methods:**

An original electronic survey consisting of 12 multiple choice questions was sent to all the members of the National Spine Surgery Society (497 surveyed). Participants were asked about their type of medical practice, years of experience in the field, society memberships, type of brace they usually prescribed, average hours of daily brace wearing they recommended, and how and when they indicated bracing discontinuation as well as the clinical and/or imaging findings this decision was based on. Exclusion criteria include brace discontinued because of having developed a curve that warranted surgical treatment.

**Results:**

Of a total of 497 surgeons, 114 responded the survey (22.9%). 71.9% had more than 5 years of experience in the specialty, and 51% mainly treated pediatric patients. Overall, 95.5% of the surgeons prescribed the thoracolumbosacral orthosis (TLSO), indicated brace wearing for a mean of 20.6 h daily. Regarding bracing discontinuation, indicated gradual brace weaning, a decision 93.9% based on anterior-posterior (AP) and lateral radiographs of the spine and physical examination, considered a Risser ≥ IV and ≥ 24 months post menarche.

**Conclusions:**

The results of this study provide insight in the daily practice of spine surgeons regarding how and when they discontinue bracing in AIS. The decision of bracing discontinuation is based on AP/lateral spinal radiographs and physical examination, Risser ≥ IV, regardless of Tanner stage, and ≥ 24 months post menarche. Gradual weaning is recommended.

## Background

When and how to discontinue bracing treatment in patients with idiopathic scoliosis who are reaching skeletal maturity and are not candidates for surgery is a historically complex issue in the management of this deformity on which little has been published. Currently, in the literature, there is little consensus either on when or how the spine surgeon should discontinue the bracing treatment in patients with adolescent idiopathic scoliosis (AIS), the imaging parameters that should be considered, whether weaning should be gradual and progressive, and based on what studies or clinical signs discontinuation should be determined [1–3].

In the absence of an objective and validated protocol, bracing control and discontinuation as well as clinical care of the patient with AIS are subjected to heterogeneous expert opinions without a standardized criteria resulting in a lack of clarity in the management of the condition. Given these limitations, it would be useful to establish a series of guidelines with the aim of improving the quality of life of the patient and reducing the cost and social burden of bracing treatment. A better understanding of bracing management based on an academic consensus of members of the National Spine Society may allow for a protocol on bracing discontinuation in AIS focusing on clinical and imaging findings.

The aim of this study was to characterize the management of bracing in AIS from the National Spine Surgery Society (SAPCV), regarding when and how to discontinue the brace in patients who are reaching skeletal maturity but are not candidates for surgical treatment through an electronic survey sent to all the members of the national society in order to evaluate the possibility of developing consensus-based guidelines.

## Methods

An original electronic survey consisting of 12 multiple choice questions was sent to all 497 members of the National Spine Surgery Society (SAPCV) (Table 1). Participants could opt for more than one answer, and if required, an additional response could be added. The demographic and clinical features of the patients that were the subject of this survey were (1) patients with idiopathic scoliosis, (2) between 10 years of age and the time of reaching skeletal maturity (adolescents), and (3) regardless of the type of brace used. Participants were asked not to include patients with curves greater than 50° or candidates for surgery or those who develop thoracic lordosis.

**Table 1:**
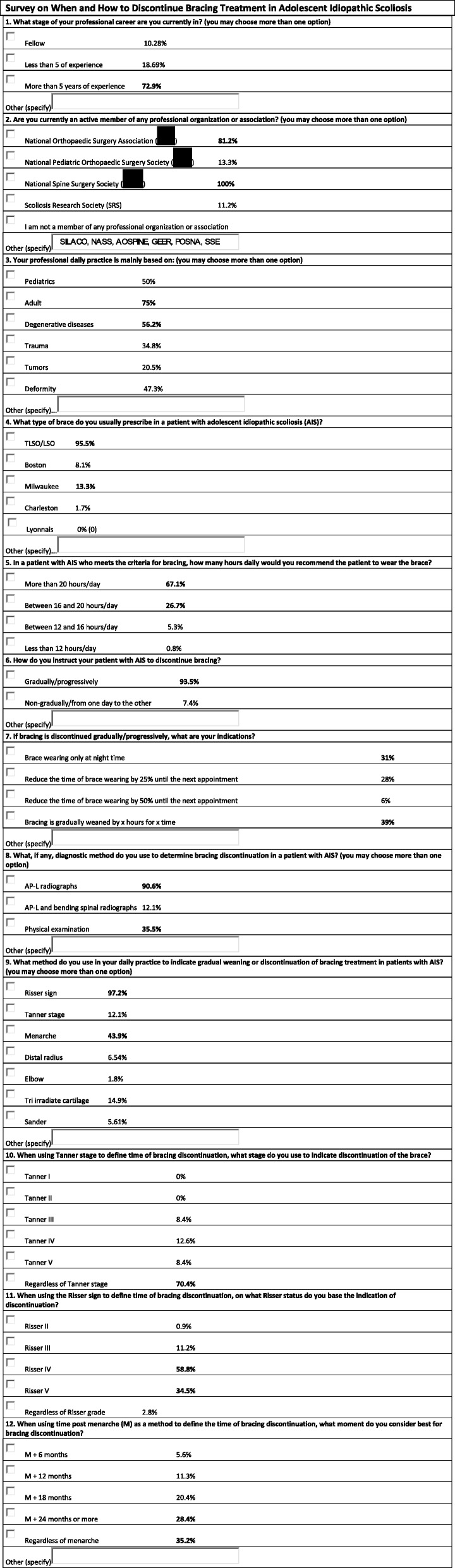
Survey on when and how to discontinue bracing treatment in adolescent idiopathic scoliosis

The variables considered in the survey were years of experience in the specialty, membership of national and international societies, and type of practice (adults, pediatrics, deformity, etc.) of the respondent, as well as the type of brace they prescribe in patients with AIS, hours of daily brace wearing recommended, and the clinical and imaging findings on which their decision to discontinue bracing treatment is based.

The survey was designed and administered using the online platform surveymonkey.com (Survey Monkey, CA, USA), and the survey was sent through an invitation by e-mail after written approval from the National Spine Society. No incentives were offered for participating in the survey.

## Results

Of a total of 497 spine surgeons who were contacted by e-mail, 114 responded the survey, with a response rate of 22.9%. Of the surveyed population, 10.2% were doing a post-residency second fellowship, 18.6% had less than 5 years of experience, and 72.9% had more than 5 years of experience in spine surgery at the moment of the survey.

Overall, 100% of the surveyed population stated to be a member of the National Spine Surgery Society (SAPCV), 81.2% of the National Orthopaedic Surgery Association (AAOT), 13.3% of the National Pediatric Orthopaedic Surgery Society (SAOTI), and 11.2% of the Scoliosis Research Society (SRS). Seventy-five percent of the surgeons stated to work mainly with adult patients, 50% with children, 47.3% specialized in deformity, 34.8% in trauma, and 20.5% in tumors. Within pediatric deformity, 75.5% of the respondents stated to have more than 5 years of experience in the field.

Overall, 95.5% of the surveyed population prescribed the thoracolumbosacral (TLSO) brace, 13.4% the Milwaukee brace, and 8% the Boston brace. When asked how many hours they recommended the patients to wear the brace, 67.1% stated to prescribe brace wearing for more than 20 h daily, 26.7% for an average of 18 h, 5.3% for an average of 14 h, and 0.9% for less than 12 h daily. Of the surgeons working in pediatric practice, 94.6% indicated wearing of the TLSO brace for an average of 20.6 h daily, without significant differences between those who had more and those who had less than 5 years of experience in the field.

Furthermore, 93.5% of the surgeons stated that, regardless of the type of brace, they discontinue bracing treatment gradually or progressively. Of those mainly working with pediatric patients, 98.2% recommend gradual discontinuation of the brace. Of the surgeons with less than 5 years of experience in the field 98.2% and of those with more than 5 years of experience 90.1% recommend gradual brace weaning from the brace.

A 39% of the respondents recommend their patients to discontinue brace wearing without a fixed time schedule and quantity of hours, until the following control visit; 31% recommend discontinuation by reducing time of brace wearing to only night time bracing until the following visit; 28% indicate their patients to reduce the time of brace wearing by 25% until the following visit; and 6% indicate the patient to reduce the time of brace wearing by 50% until the following visit. Of the surgeons working with pediatric patients, 34.8% indicate their patients to reduce bracing according to their convenience (without a fixed time schedule and quantity of hours) until the following control visit, 30.4% recommend brace wearing only at night time, 28.3% indicate to reduce brace wearing by 25% and 6.5% by 50% until the following visit. Of those with less than 5 years of experience, 54% recommend only night time brace wearing and of those with more than 5 years of experience 34.2% recommend to reduce brace wearing to night time only and 34.2% recommend their patients to discontinue brace wearing according to their own convenience until the following visit.

Regarding clinical and imaging findings on which discontinuation was based, 90.6% of the surveyed population based their decision on posterior-anterior and lateral radiographs, 85.5% on radiographs and clinical findings, and 35.3% on clinical findings only. Of the surgeons who work in pediatric practice, 93.9% based their decision to discontinue bracing treatment on a combination of radiographs and clinical examination. Of the surgeons with less than 5 years of experience in the field, 66.7% based their decision of brace discontinuation on anterior-posterior and lateral radiographs and of those with more than 5 years of experience on both anterior-posterior and lateral radiographs and physical examination.

When evaluating the role of clinical findings in the decision-making on bracing treatment discontinuation, 97.2% of the surveyed population considered the Risser sign, 43.9% menarche, 14.9% the tri irradiate cartilage, and 12.1% Tanner stage, among others, to be important signs. When breaking up these data, 58.8% use a Risser sign ≥ IV. Overall, 70.4% stated they indicated discontinuation of the orthosis regardless of Tanner stage, regardless of menarche 35.2% and 28.4% ≥ 24 months post menarche.

Among surgeons working in pediatric practice, 93.3% recommend brace discontinuation at a Risser ≥ IV, 63.6% regardless of Tanner stage, and 36.5% at 18 months post menarche. Of the surgeons with less than 5 years of experience, 93.6% recommended bracing discontinuation at Risser sign ≥ IV, 42.8% regardless of Tanner stage, 50% ≥18 months post menarche, while of those with more than 5 years of experience, 98.8% recommend discontinuation at Risser ≥ IV, 69.2% regardless of Tanner stage, and 48.8% ≥ 24 months post menarche.

The results of the survey were analyzed, and a final list of recommendations was established (Table 2).

**Table 2:** Study variables on which the highest degree of consensus was reached (%) in pediatric spine surgeons

Gradual weaning from the brace	98.2%
AP/L radiographs + physical exam	93.9%
Risser ≥ IV	93.3%
Risser + menarche	87.5%
Use of the brace > 20 h/day	68.7%
Regardless of Tanner stage	63.6%
Weaning (*x* hours for *x* time)	54.1%
Brace discontinuation according to menarche > 24 months	48.8%

## Discussion

To our knowledge, currently there is little consensus in the literature on how and when to discontinue bracing treatment in AIS [2, 3]. In one of the few studies published in the literature, Andersen et al. administered a questionnaire to 136 patients with AIS who underwent bracing treatment and found that daily activities and social contacts were affected during treatment and follow-up. They concluded that brace weaning should be started as early as possible and not later than 36 months post menarche in girls [3].

In another interesting and well-known published paper, Negrini et al. among the SOSORT Society made recommendations about the weaning brace issue in the 2016 SOSORT Guidelines. The stated that “It is recommended that braces are worn until the end of vertebral growth and then the wearing time is gradually reduced…” and the second recommendation was…“It is recommended that the wearing time of the brace is gradually reduced while performing exercises...” [4]. We believe that these treatment guidelines should be taken into account when performing or conducting any spine deformity non-surgical basic study. Take into consideration that there is no straight age correlation about stature growth and bone mass density in published literature. Any kind of aerobic or anaerobic exercises are recommended during brace treatment.

This electronic survey was conducted with the aim to characterize the management of bracing discontinuation among members of the National Spine Society. They were asked for their expert opinion on this issue in an attempt to develop guidelines regarding brace weaning in patients with AIS with a special focus on the variables listed in Table 2.

The opinions of the surgeons, however, cannot be generalized as they develop their activities in different areas within the field of spine surgery. Additionally, it would be difficult to determine whether this expert opinion may modify the decision-making of spine surgeons regarding brace discontinuation in these patients.

One of the weaknesses of our study is the limited number of variables evaluated in the survey as other findings that were not mentioned may have to be included in working guidelines for bracing discontinuation. An additional limitation may be that the surveyed population consisted of spine surgeons from a single country with a relatively low response rate (22.9%). Another weakness of the study is that participants were not asked how much time apart they scheduled control visits and on what parameters they based this decision. Nevertheless, one of the strengths of the study is the large population surveyed—a total of 114 surgeons responded—and the fairly homogeneous nature of their responses. Another strength of the study is that all the members of the National Spine Surgery Society were included, of whom a considerable percentage felt compelled to answer the survey in spite of not specializing in pediatric deformity. The methodology of an electronic survey allowed us to reach all the members of the NSSS while ensuring anonymity and thereby avoiding of bias. The electronic modality of the survey facilitated fast and reliable data collection. It should be taken into account that this methodology provides an overview of expert opinion rather than validated scientific evidence.

Currently, there is no standardized strategy to determine in this extremely crucial matter of how and when to discontinue bracing in AIS and a standardized protocol and algorithm should be developed.

Future validation studies would be necessary to define whether the inclusion of the variables evaluated in this survey are useful in the daily practice of spine surgeons treating patients with AIS, including a larger number of participants with involvement international societies. Clinical and imaging variables are important in the decision-making on bracing discontinuation in AIS. The indication and management of bracing discontinuation should be based on continuous interaction between the opinion of surgeon and the needs and objectives of the patient.

## Conclusion

Based on the survey, the main variables considered in the management of bracing discontinuation in AIS were gradual brace weaning, AP-L radiographs together with physical examination, Risser status ≥ IV, regardless of Tanner stage, ≥ 24 months post menarche, and a weaning method (*x* hours for *x* time).

The results of the study help to shed a light on the decision-making in the management of non-surgical treatment of children with AIS by the spine surgeon, which may be useful when outlining consensus-based working guidelines for the management and discontinuation of bracing in patients with AIS.

## References

[1] Weinstein SL, Dolan LA, Wright JG (2013). Effects of bracing in adolescents with idiopathic scoliosis. N Engl J Med.

[2] Steen H, Lange JE, Brox JI (2015). Early weaning in idiopathic scoliosis. Scoliosis.

[3] Andersen MO, Andersen GR, Thomsen K (2002). Early weaning might reduce the psychological strain of Boston bracing: a study of 136 patients with adolescent idiopathic scoliosis at 3.5 years after termination of brace treatment. J Pediatr Orthop B.

[4] Negrini S, Donzelli S, Aulisa AG (2018). 2016 SOSORT guidelines: orthopaedic and rehabilitation treatment of idiopathic scoliosis during growth. Scoliosis Spinal Disord.

